# Identification, Genome Characterization, and Growth Optimization of *Paenibacillus peoriae* MHJL1 for Biocontrol and Growth Promotion of Cotton Seedlings

**DOI:** 10.3390/microorganisms13020261

**Published:** 2025-01-24

**Authors:** Tongtong Zheng, Min Li, Zhengnan Kong, Lei Ji, Xiaowen Fu, Li Dai, Jizhen Kan, Qingyong Men, Hailong Wang, Binghai Du, Kai Liu, Xiangui Mei, Chengqiang Wang

**Affiliations:** 1 National Engineering Research Center for Efficient Utilization of Soil and Fertilizer Resources, Shandong Engineering Research Center of Plant-Microbia l Restoration for Saline-Alkali Land, Shandong Key Laboratory of Agricultural Microbiology, College of Life Sciences, Shandong Agricultural University, Tai’an 271018, China; 18453782137@163.com (T.Z.); m17866707958@163.com (M.L.); 15552817006@163.com (Z.K.); daili@sdau.edu.cn (L.D.); du_binghai@163.com (B.D.); liukai_1982@163.com (K.L.); 2 Shandong Provincial Key Laboratory of Applied Microbiology, Ecology Institute, Qilu University of Technology (Shandong Academy of Sciences), Jinan 250103, China; jilei.1010@163.com (L.J.); seanfv@163.com (X.F.); 3 Juxian Agricultural Technical Service Center, Rizhao Academy of Agricultural Science, Rizhao 276800, China; weizhixuna@163.com (J.K.); jxnyjfzghk@rz.shandong.cn (Q.M.); 4 State Key Laboratory of Microbial Technology, Institute of Microbial Technology, Shandong University, Qingdao 266237, China; wanghailong@sdu.edu.cn

**Keywords:** *Paenibacillus peoriae*, cotton, whole-genome analysis, fusaricidins, microbial fertilizer

## Abstract

Fusarium and verticillium wilt are the primary diseases affecting cotton plants, significantly reducing both the yield and quality of cotton. *Paenibacillus* spp. are crucial biocontrol strains for controlling plant diseases. In this study, *Paenibacillus peoriae* MHJL1, which could prevent the pathogenic fungi of fusarium and verticillium wilt and promote cotton growth, was isolated from the rhizosphere soil of cotton plants. Whole-genome analysis of strain MHJL1 identified 16 gene clusters for secondary metabolite synthesis, including fusaricidins with potent antifungal properties. By optimizing the fermentation process, the cell and spore numbers of MHJL1 were increased to 2.14 × 10^8^ CFU/mL and 8.66 × 10^8^ CFU/mL, respectively. Moreover, the antifungal ability of MHJL1 was also increased by 31.48%. In pot experiments conducted with healthy soil, the control rates for MHJL1 against fusarium and verticillium wilt were found to be 44.83% and 58.27%, respectively; in experiments using continuously cropped soil, the control rates were 55.22% against fusarium wilt and 48.46% against verticillium wilt. Our findings provide valuable insights for the biocontrol application and fermentation of *P. peoriae* MHJL1, while also contributing a new resource for the development of microbial agents.

## 1. Introduction

Cotton is a crucial economic crop grown in various countries and regions around the world [1]. Cotton crops suffer from more than 40 types of disease during their growth and development, among which, fusarium and verticillium wilt are significant challenges for the industry. Additionally, pathogens of fusarium and verticillium wilt often occur together, which severely affect the yield and quality of cotton [2,3,4,5]. Fusarium and verticillium wilt are primarily caused by *Fusarium oxysporum* and *Verticillium dahliae*, respectively. Both pathogens infect the vascular bundles of cotton plants, obstructing the vessels and hindering the absorption of water and nutrients. The infection can occur at any stage of the plant’s development [6,7].

Currently, chemical control is the primary method used to treat fusarium and verticillium wilt in cotton plants. However, this approach has limitations. Chemical treatments primarily address diseases affecting the above-ground portions of the plants, which means their effectiveness against soil-borne diseases caused by below-ground pathogens is limited. Furthermore, the long-term use of chemical treatments can lead to the development of resistance in pathogens and may negatively impact environmental microbial diversity [8]. As a result, biological control using plant-growth-promoting rhizobacteria (PGPR) has emerged as a significant area of research. This method of biological control is economical, safe, non-toxic, and provides long-lasting effects, making it a popular choice for managing soil-borne diseases [9].

As a category of PGPR used for biological control, *Paenibacillus* species are sourced from a variety of origins, possess strong reproductive capabilities, and are characterized by their non-toxic properties. Among these species, *P. peoriae* is recognized for its beneficial biocontrol functions. For instance, *P. peoriae* RhAn32 can inhibit the growth of *Fusarium verticillioides* and exhibit a promoting effect on plants [10], *P. peoriae* SP9 has significant application potential in bactericidal performance and maintaining the microecological balance of soil [11], *P. peoriae* To99 has been reported to inhibit bacterial spot caused by *Xanthomonas* [12], *P. peoriae* ZBSF16 can produce iron carriers and reduce the infection rate and disease index of grape white rot [13], and the growth parameters of pines treated with *P. peoriae* strain HJ-2 were significantly higher than those of the control group, and the incidence of stem rot was notably decreased in both greenhouse and field conditions [14]. However, research into the biocontrol functions of *P. peoriae* remains limited, and there have been no reports on its application in controlling cotton diseases.

In this study, *P. peoriae* MHJL1 was isolated from Jining City, Shandong Province, China. It demonstrated antagonistic effects in controlling pathogens responsible for fusarium and verticillium wilt in cotton. The identification of this strain was based on morphological, physiological, and biochemical characteristics, as well as genome analysis. The antimicrobial substances produced by *P. peoriae* MHJL1 were analyzed preliminarily, and the fermentation process was optimized. Additionally, pot experiments were conducted on cotton seedlings. This research highlights the significant potential for the industrial fermentation and biocontrol applications of MHJL1.

## 2. Materials and Methods

### 2.1. Experimental Materials

The rhizosphere soil samples of cotton plants, which were used to screen PGPR in this study, were obtained from Jining City, Shandong Province, China. The cotton variety used in this experiment was No. 63, which had a certain resistance to fusarium and verticillium wilt, and it was purchased from Qingfeng Seed Industry Co., Ltd. (Dezhou, China). Pathogenic fungi *F. oxysporum* and *V. dahliae* were provided by the Chinese Academy of Agricultural Sciences [15,16].

### 2.2. Media

Luria–Bertani medium (LB), potato dextrose agar medium (PDA), and bean sprout juice were used to culture the bacteria and fungi and optimize the media, respectively, and were prepared according to the reported reference [17,18]. The YS-1 medium was used to test the inhibition effect and was prepared as reported [19].

### 2.3. Screening and Identification of the Rhizosphere PGPR of Cotton Seedlings

The agar plate dilution method was employed to isolate rhizosphere strains [20]. A total of 10 g of rhizosphere soil was taken and added to 90 mL of sterile water with an appropriate amount of glass beads in a flask to shake for 30 min. The soil suspension was diluted into different concentration gradients, and 0.1 mL of the soil suspension from each gradient was evenly spread on LB. The strains on the LB media were then incubated at 37 °C for 1–3 days. Once colonies grew on the media, single colonies were purified, picked, and transferred to new media, numbered, and stored at 4 °C for future use. The plate confrontation experiment [20] was used to screen the PGPR controlling *F. oxysporum* and *V. dahliae*. The inhibition zones were measured to calculate the inhibition rates, and PGPR that showed significant and stable effects were selected.

In accordance with the methodology outlined in [21], the genomic DNA of PGPR was extracted, and the sequences of 16S rDNA were amplified using 27F/1492R primers and sent to Sangon Biotech (Shanghai, China) for sequencing. The sequencing results were compared and analyzed with relevant sequences from the NCBI database, and phylogenetic trees were constructed using the neighbor-joining method of Mega 5.0 [22] to identify PGPR.

### 2.4. Whole-Genome Analysis of Strain MHJL1

The genomic DNA of strain MHJL1 was extracted and then sent to Shanghai Personalbio Technology Co., Ltd. (Shanghai, China) for genome sequencing. The PacBio Sequel (PacBio, Menlo Park, CA, USA) and Illumina NovaSeq (Illumina, Inc., San Diego, CA, USA) sequencing platforms were used to sequence. The data obtained by PacBio were assembled with HGAP 2.3 and CANU v1.5 software to obtain contig sequences [23,24]. The data obtained via Illumina Nova 6000 were used to correct the above contig sequences, and the complete sequence of strain MHJL1 was finally spliced [25].

The whole-genome analysis of MHJL1 was conducted with NR, Swiss-Prot, TCDB, Pfam, COG, GO, and KEGG databases to annotate the gene function. Then, PHI (Pathogen Host Interactions Database), VFDB (Virulence Factors of Pathogenic Bacteria), and CARD (Comprehensive Antibiotic) were used to predict the virulence and drug-resistance genes, and antiSMASH was used to predict the gene clusters responsible for coding the synthetic enzymes of secondary metabolites [26]. For the assembled genome sequence, the results of the predicted coding genes, non-coding RNAs, and gene function annotations were combined, and the genome of strain MHJL1 was visualized using Circos 0.62 software [27].

### 2.5. Identification and Analysis of Antimicrobial Substances in the Fermentation Broth of Strain MHJL1

The fermentation broth of strain MHJL1 was extracted by adding an equal volume of n-butanol and then concentrated according to the reported method [28]. After filtration with a 0.22 μm organic membrane, the identification and analysis of antimicrobial substances were performed via liquid chromatography tandem–mass spectrometry (LC-MS/MS) according to the reported method, and a C18 column was used to determine the concentration of the antimicrobial substances with two mobile phases of A (0.1% acetic acid in water) and B (0.1% acetic acid in acetonitrile) [29]. MS-DIAL 5 was used to analyze the data, and the specific products were identified according to the MS-DIAL database of biological standards [30].

### 2.6. Fermentation Process Optimization of Strain MHJL1

Strain MHJL1 was inoculated on an LB plate for activation, and single colonies were selected and inoculated into triangular flasks containing 30 mL of liquid medium and then incubated at 37 °C and 180 rpm for 12 h to create the seed solution.

Using bean sprout juice as the base culture medium, the components of the fermentation medium for culturing MHJL1 were optimized through single-factor and response surface methodologies [31]. Various carbon sources, including soluble starch, potato powder, sucrose, glucose, and corn flour, were tested at concentrations of 10 g/L, 20 g/L, 30 g/L, 40 g/L, and 50 g/L, respectively. Additionally, organic nitrogen sources such as yeast powder, peptone, rapeseed meal powder, and soybean meal powder were evaluated at concentrations of 10 g/L, 15 g/L, 20 g/L, and 25 g/L, respectively. Inorganic nitrogen sources, comprising ammonium sulfate, ammonium chloride, and potassium nitrate, were assessed at concentrations of 2 g/L, 4 g/L, 6 g/L, and 8 g/L, respectively. Furthermore, inorganic salts, including magnesium sulfate, calcium carbonate, dipotassium hydrogen phosphate, and monopotassium phosphate, were utilized at concentrations of 1 g/L, 2 g/L, 3 g/L, 4 g/L, and 5 g/L, respectively. The cultural conditions were also optimized for culturing strain MHJL1. The fermentation conditions of seed age, inoculum size, liquid volume, initial pH, rotation speed, and temperature were optimized. The seed ages were set at 8 h, 10 h, 12 h, 14 h, and 16 h, respectively. The initial pH values were set at 5, 6, 7, and 8, respectively. The temperatures were set at 28 °C, 32 °C, 37 °C, and 42 °C, respectively. The rotation speeds were set at 160 rpm, 180 rpm, 200 rpm, and 220 rpm, respectively. The inoculum concentrations were established at 1%, 2%, 3%, 4%, and 5%, respectively. The liquid volumes in flasks of 250 mL were set to 25 mL, 50 mL, 75 mL, and 100 mL, respectively, with 2% seed liquid inoculation. The principle of single-factor analysis was employed throughout the study. The optimal process was determined using the agar plate dilution method for colony counting.

The optimization of medium components for increasing the spore amount of strain MHJL1 was conducted using the above-optimized fermentation medium. The concentrations of carbon sources were varied at levels of 30 g/L, 38 g/L, 45 g/L, 50 g/L, 55 g/L, and 60 g/L, respectively. Organic nitrogen sources were tested at concentrations of 15 g/L, 20 g/L, 24 g/L, 30 g/L, and 35 g/L, respectively. Inorganic nitrogen sources were adjusted to levels of 0.5 g/L, 1 g/L, 2 g/L, 4 g/L, 6 g/L, and 8 g/L, respectively. Additionally, the concentrations of inorganic salts were set at 1 g/L, 2 g/L, 3 g/L, 3.9 g/L, 5 g/L, and 6 g/L, respectively. The cultural conditions were also optimized for increasing the spore amount of MHJL1 as for the living cell optimization. The seed ages were set at 8 h, 10 h, 12 h, 14 h, and 16 h, respectively. The initial pH values were set at 5, 6, 7, 8, and 9, respectively. The temperatures were set at 30 °C, 32 °C, 35 °C, 37 °C, and 40 °C, respectively. The rotation speeds were set at 140 rpm, 160 rpm, 180 rpm, 200 rpm, and 220 rpm, respectively. The inoculum concentrations were established at 1%, 2%, 3%, 4%, and 5%, respectively. The liquid volumes in flasks of 250 mL were set to 25 mL, 50 mL, 75 mL, and 100 mL, respectively, with 2% seed liquid inoculation. The principle of single-factor analysis was always maintained, with the initial pH being natural, a liquid volume of 50 mL, and 2% amount of seed liquid. The stirring speed was set at 180 rpm, the temperature was set at 37 °C, and the shaking cultivation was prolonged for 36 h. After a water bath at 80 °C, the spore number was determined using the agar plate dilution technique.

The fermentation optimization of the production of antifungal substances by MHJL1 was conducted using YS-1 as the base medium. Different carbon sources (corn flour, potato flour, sucrose, glucose, and soluble starch at concentrations of 50 g/L, 60 g/L, 70 g/L, 80 g/L, and 90 g/L, respectively) and organic nitrogen sources (urea, soybean meal, peptone, rapeseed meal, and yeast powder at concentrations of 10 g/L, 15 g/L, 20 g/L, 25 g/L, and 30 g/L, respectively) were selected for testing, while maintaining the principle of a single factor. A total of 2% seed liquid was inoculated into 50 mL of sterilized medium and cultured in a shaking flask at 180 rpm and 37 °C. Samples were taken at 0 h, 8 h, 12 h, 24 h, 36 h, 48 h, 60 h, 72 h, 84 h, and 96 h, and the control activity of the fermentation broth was also measured. The Oxford cup method was used as the measurement method [32,33]. Each of the above treatments was repeated three times to determine the optimal composition.

### 2.7. Pot Experiments for Controlling the Fusarium and Verticillium Wilt of Cotton Using Strain MHJL1

The pathogens of the fusarium and verticillium wilt of cotton were inoculated onto solid plates of potato dextrose agar (PDA) and cultured at 28 °C for 7 days. After that, hyphae were inoculated into 50 mL of PDA liquid media. The pathogens were shaken at 28 °C and 160 rpm for 48 h, and then filtered through a sterile filter. The bacterial amount of the microbial suspension was adjusted to 6 × 10^6^ CFU/mL. The healthy soil was obtained at the experimental base of Shandong Agricultural University, and the continuously cropped soil of cotton was obtained from Jining City, Shandong Province, China. The raw soil was mixed with vermiculite. Six groups were set up for the pot experiments: the control group (CK) containing only water; a group containing the fermentation liquid of strain MHJL1; a group containing the fermentation liquid of the pathogen *V. dahliae*; a group containing the fermentation liquid of the pathogen *F. oxysporum*; a group containing both the fermentation liquids of *V. dahliae* and strain MHJL1; and a group containing both the fermentation liquids of *F. oxysporum* and strain MHJL1. Three distinct biological treatments were set for each group. Following the planting process, the status of cotton seedlings was monitored every day. When diseases were stabilized, the incidence of cotton seedlings under different treatment conditions was statistically analyzed. The disease levels were recorded according to the following classification criteria, and a disease index was calculated to determine the relative control effectiveness. Classification standards: Level 0: cotton seedlings are healthy and grow normally, with no diseased leaves; Level 1: the diseased area is less than one-third of the total area of cotton seedlings; Level 2: the diseased area is more than one-third but less than two-thirds of the total area of cotton seedlings; Level 3: the diseased area is more than two-thirds of the total area of cotton seedlings; Level 4: the cotton seedlings are severely wilted or dead. Disease index = (∑level number × number of diseased plants at each level)/(total number of surveyed plants × highest disease level) × 100. Control effect (%) = (control disease index − treatment disease index)/control disease index × 100. The averages and the standard deviations of the data are presented.

### 2.8. Data Statistics and Analysis

SPSS statistical software version 19.0 (IBM, Armonk, NY, USA) was used to analyze the data results via a one-way analysis of variance (ANOVA) with a Duncan’s multiple range test; meanwhile, each value was represented by the mean ± standard deviation, and different superscripts showed a significant difference (*p* < 0.05) [4]. Figures are presented using Origin software 2022 (OriginLab Corp., Northampton, MA, USA) [20].

## 3. Results

### 3.1. Screening, Functional Analysis, and Identification of P. peoriae MHJL1

**Figure 1:**
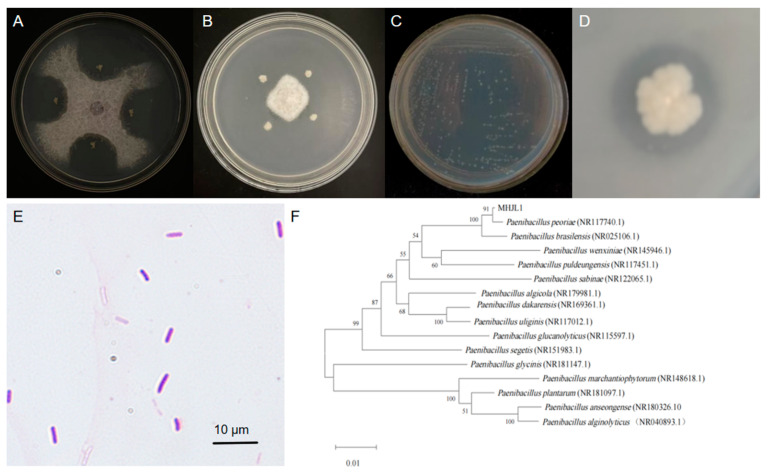
Identification and functional analysis of strain MHJL1. (**A**) The inhibitory effect of MHJL1 on *F. oxysporum*. (**B**) The inhibitory effect of MHJL1 on *V. dahlia*. (**C**) The colony morphology of MHJL1. (**D**) The growth characteristics of MHJL1 on a bacterial medium with organic phosphorus; a transparent circle appeared. (**E**) The cell morphology (magnification: 10 × 100) of MHJL1. (**F**) The phylogenetic tree of MHJL1 based on 16S rRNA.

In this study, PGPR from cotton rhizosphere soils were screened to control the pathogenic fungi *F. oxysporum* and *V. dahlia*. Among the isolated PGPR, the strain MHJL1 showing inhibitory effects on both pathogenic fungi (Figure 1A,B) was obtained. Colonies of MHJL1 on LB solid medium are round, form a flat dry surface and spiny edge, and show a milky translucent color (Figure 1C). The cells of MHJL1 are rod-shaped and Gram-positive (Figure 1E). The results of physiological and biochemical tests indicated that MHJL1 was positive for mannitol, xylose, arabinose, starch hydrolysis, gelatin liquefaction, glucose, and nitrate reduction and organophosphorus dissolving (Figure 1D), while being negative for oxidase, catalase, citrate utilization, indole, and hydrogen sulfide.

The 16S rRNA sequence of MHJL1 was analyzed, and a phylogenetic tree was generated, as shown in Figure 1F. The result indicated that MHJL1 was closely related to *P. peoriae*. Combining the morphological, physiological, and biochemical reaction characteristics, as well as the phylogenetic analysis results based on the 16S rRNA sequence, strain MHJL1 was preliminarily identified as *P. peoriae*.

**Figure 2:**
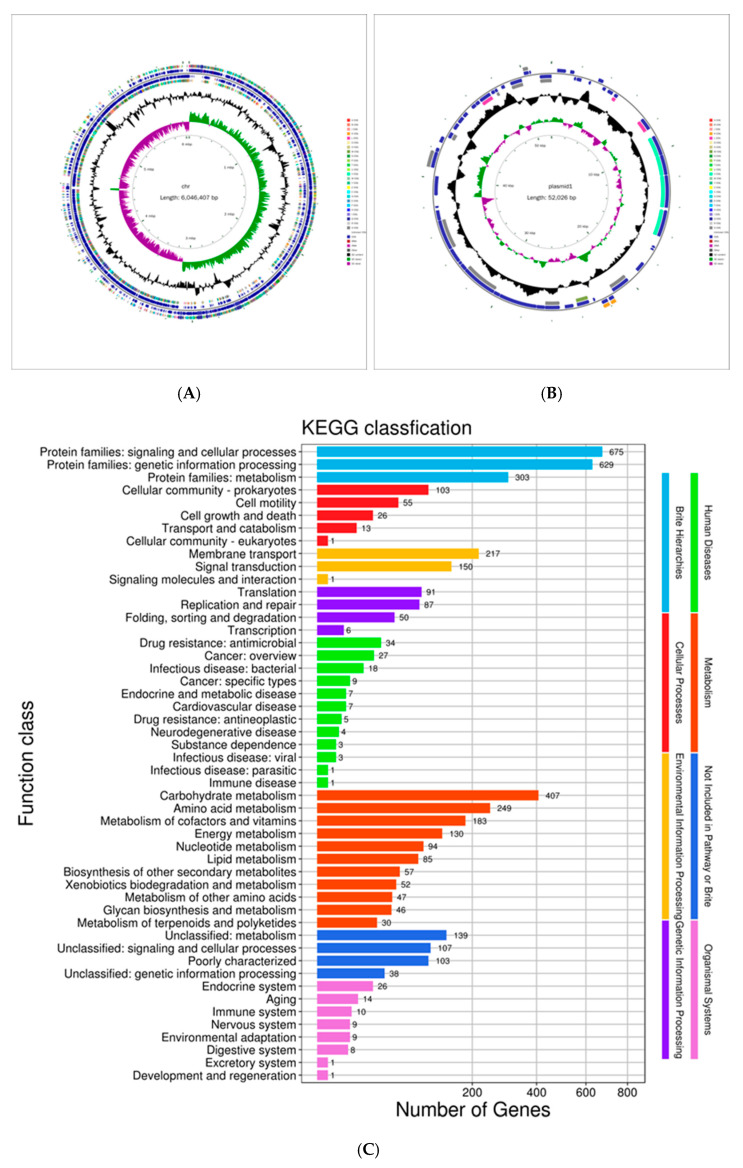
The maps and genome annotation of strain MHJL1. (**A**) The circular chromosome map of MHJL1. (**B**) The circular plasmid map of MHJL1. (**C**) The KEGG classification of the genes identified within MHJL1.

To further elucidate the taxonomic classification of MHJL1 and explore its genetic basis for controlling disease and promoting growth, the whole genome of strain MHJL1 was sequenced and the sequence was deposited in the National Center for Biotechnology Information (NCBI) under GenBank accession numbers CP157265.1 and CP157266.1.1. The sequencing results showed that strain MHJL1 contained a circular chromosome with a sequence length of 6,046,407 bp and a GC content of 46%, as well as a circular plasmid with a sequence length of 52,026 bp and a GC content of 41% (Figure 2A,B). ANI analysis was performed between MHJL1 and the related species. Strain MHJL1 had the highest ANI value with *P. peoriae*, reaching 95%, and the corresponding coverage rate was 87%. Thus, strain MHJL1 was finally identified as *P. peoriae*.

A total of 5599 genes were predicted in the complete genome of MHJL1. In the chromosome genome, there were 210 non-coding RNAs, including 39 rRNAs, 107 tRNAs, and 64 other non-coding RNAs; no non-coding RNAs were found in the plasmid. The gene annotation results of strain MHJL1 using the KEGG database are shown in Figure 2C. A total of 4371 genes were annotated and divided into 46 functional classes. These 46 classes were associated with different metabolic pathways and divided into seven major categories: simplified hierarchical structure, cellular processes, environmental information processing, genetic information processing, human diseases, metabolism, and biological systems. It is important to highlight that strain MHJL1 possesses 16 gene clusters associated with the synthesis of secondary metabolites. Notably, three of these gene clusters exhibit 100% similarities to the established gene clusters responsible for coding the synthetic enzymes of fusaricidin B, paenilan, and tridecaptin, respectively.

### 3.2. Identification and Analysis of Antimicrobial Substances in the Fermentation Liquid of Strain MHJL1

The antibiotics detected in the fermentation liquid of strain MHJL1 are shown in Table 1. A series of fusaricidins that could control pathogenic fungi were successfully detected: a substance with a mass-to-charge ratio of 883 Da ([M + H]^+^) and a molecular formula of C_41_H_74_N_10_O_11_ was presumed to be the signal of fusaricidin A; a substance with a mass-to-charge ratio of 897 Da ([M + H]^+^) and a molecular formula of C_42_H_76_N_10_O_11_ was presumed to be the signal of fusaricidin B; a substance with a mass-to-charge ratio of 947 Da ([M + H]^+^) and a molecular formula of C_45_H_74_N_10_O_12_ was presumed to be the signal of fusaricidin C; a substance with a mass-to-charge ratio of 961 Da ([M + H]^+^) and a molecular formula of C_46_H_76_N_16_O_14_ was presumed to be the signal of fusaricidin D; a substance with a mass-to-charge ratio of 911 Da ([M + H]^+^) and a molecular formula of C_43_H_78_N_10_O_11_ was presumed to be the signal of lipopeptide antibiotic LI-F05b/LI-F08a; a substance with a mass-to-charge ratio of 931 Da ([M + H]^+^) and a molecular formula of C_45_H_74_N_10_O_11_ was presumed to be the signal of lipopeptide antibiotic LI-F07a; and a substance with a mass-to-charge ratio of 945 Da ([M + H]^+^) and a molecular formula of C_46_H_76_N_10_O_11_ was presumed to be the signal of lipopeptide antibiotic LI-F07b.

### 3.3. Optimization of the Fermentation Process for Strain MHJL1

Using bean sprout juice as the base culture medium, the components of the fermentation medium for culturing strain MHJL1 were optimized to contain 38 g/L potato powder, 24 g/L soybean meal powder, 3.8 g/L magnesium sulfate, and 4 g/L ammonium chloride. Furthermore, the best fermentation conditions were an inoculum age of 12 h, 3% inoculum amount, 50 mL/250 mL liquid volume, pH 6, fermentation temperature of 37 °C, and shaking speed of 200 rpm. As a result, the cell number of strain MHJL1 could reach 2.14 × 10^8^ CFU/mL via the optimized fermentation process (Supplementary Materials—Figures S1–S3).

Based on the cell number optimization of living cells, the best fermentation medium for spore production was also determined to contain 60 g/L potato powder, 24 g/L soybean meal powder, 1.94 g/L magnesium sulfate, and 1 g/L ammonium chloride. Furthermore, the optimal fermentation conditions for spore production were a seed age of 12 h, 4% inoculum amount, 50 mL/250 mL liquid volume, pH 6, fermentation temperature of 37 °C, and shaking speed of 200 rpm, resulting in an increase in spore amount from 2.41 × 10^7^ CFU/mL to 8.66 × 10^8^ CFU/mL (Supplementary Materials—Figures S4–S7).

Based on the YS-1 medium, the fermentation medium for producing antifungal substances was optimized to contain 62.28 g/L glucose, 26.37 g/L soybean meal powder, 6 g/L sodium chloride, 2 g/L magnesium sulfate, and 0.1 g/L dipotassium hydrogen phosphate. Through medium optimization, the inhibition zone diameter of strain MHJL1 was increased by 31.48% compared to the control. (Supplementary Materials—Figures S8 and S9).

### 3.4. Control Effects of Strain MHJL1 on the Fusarium and Verticillium Wilt of Cotton

**Figure 3:**
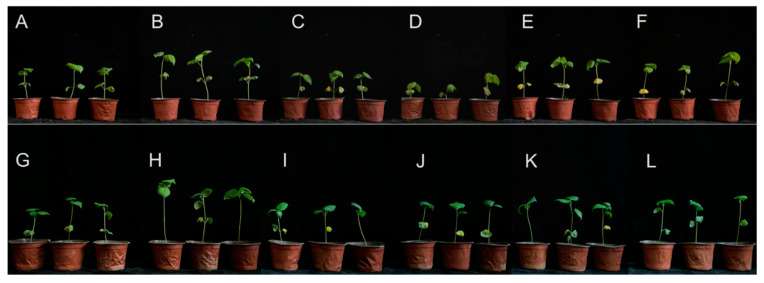
The potted cotton plants grown for 50 d in healthy soil or continuously cropped soil. (**A**–**F**) The cotton plants grown in healthy soil for 50 d. (**G**–**L**) The cotton plants grown in soil with continuously cropped obstacles for 50 d. (**A**,**G**) represent the control groups using water; (**B**,**H**) represent the microbial agent groups using the fermentation liquid of strain MHJL1; (**C**,**I**) represent the *V. dahliae* groups with only the application of *V. dahliae*; (**D**,**J**) represent the *F. oxysporum* groups with only the application of *F. oxysporum*; (**E**,**K**) represent the *V. dahliae* + microbial agent groups with the simultaneous application of *V. dahliae* and the microbial agent of strain MHJL1; (**F**,**L**) represent the *F. oxysporum* + microbial agent groups with the simultaneous application of *F. oxysporum* and the microbial agent of strain MHJL1.

The effects of the solution of strain MHJL1 on controlling the fusarium and verticillium wilt of cotton were tested. In the pot experiments with healthy soil, the relative inhibitory effect of MHJL1 against fusarium and verticillium wilt was 44.83% and 58.27%, respectively (Table 2). In the pot experiments with continuously cropped soil, the relative inhibitory effect of MHJL1 against fusarium and verticillium wilt was 55.22% and 48.46%, respectively (Table 2). The disease indices of cotton seedlings in all the treatment groups that added the microbial agent of MHJL1 were decreased compared to the control group. The *F. oxysporum* and *V. dahliae* groups, which were only inoculated with *F. oxysporum* and *V. dahliae*, respectively, showed significant differences in disease occurrence compared to those simultaneously treated with MHJL1. The findings of our study demonstrated that the pathogenic fungi exhibited a measurable degree of pathogenicity toward cotton plants, while MHJL1 was found to be effective in controlling the pathogenic fungi, thereby diminishing their associated pathogenicity (Figure 3).

For healthy soil, compared to the control, the treatment group with only the strain MHJL1 showed an increase in cotton plant height, root length, stem diameter, and fresh weight by 30%, 14.12%, 16.02%, and 24.46%, respectively; for continuously cropped soil, the increases in cotton plant height, root length, stem diameter, and fresh weight were 31.50%, 34.29%, 28.23%, and 120.47%, respectively (Table 3). The treatment group that applied both strain MHJL1 and the pathogenic fungi showed improvements in cotton plants compared to the control group or the groups that applied only the pathogenic fungi. The findings demonstrated that strain MHJL1 exhibited a positive influence on the growth of cotton (Figure 3).

## 4. Discussion

Using PGPR to suppress plant pathogens is a key strategy in biological control. In this study, *P. peoriae* MHJL1 was screened, identified, and confirmed to control the fusarium and verticillium wilt of cotton. In pot experiments using healthy soil and soil with continuously cropped obstacles, the relative control effects of MHJL1 against the fusarium and verticillium wilt of cotton were both high. Some other studies have also found that *P. peoriae* had inhibitory effects on various plant pathogens: *P. peoriae* ZF390 was reported to have a significant control effect on soft rot disease [34]; four strains of *P. peoriae* from the rhizosphere of lettuce crops were found to promote crop growth and reduce the incidence of wilt disease under field conditions [35]. Thus, it was verified that *P. peoriae* is an important species for the biological control of many plant pathogens.

In the genome of strain MHJL1, a total of 16 secondary metabolite gene clusters were discovered, and 3 gene clusters had 100% similarity to the verified synthetic gene clusters for coding the synthetic enzymes of fusaricidin B, paenilan, and tridecaptin. The three above-mentioned secondary metabolites have also been studied in some *Paenibacillus* spp., for example, *Paenibacillus polymyxa* [36]. Based on 16S rRNA, *P. peoriae* is very closely related to *P. polymyxa* and also has similar synthetic antimicrobial substances [12]. Seven homologous compounds of fusaricidins were tested via LC-MS/MS in the fermentation liquid of strain MHJL1. Fusaricidins are lipopeptide antibiotics, which have outstanding antifungal activities on fungal pathogens, such as *F. oxysporum* and *Botrytis cinerea* [37,38].

The optimization of the fermentation parameters is an important step in the successful industrialization of microbial fermentation products. It was reported that the yield of the antifungal substances of *P. polymyxa* DS-R5 was increased by 123% through an optimized culture and formula [39]. In this experiment, the cell number, spore amount, and antibiotic yield of *P. peoriae* MHJL1 were optimized. The effects of the fermentation components and conditions of strain MHJL1 were investigated via single-factor and response surface methodologies. The optimized cell number of MHJL1 could reach 2.14 × 10^8^ CFU/mL, and the amount of spores could reach 8.66 × 10^8^ CFU/mL. Furthermore, the inhibition zone diameter of MHJL1 was increased by 31.48%. In addition, the optimized organic nitrogen source was soybean meal, which is an industrial and agricultural by-product with a low cost and wide source suitable for the industrial fermentation of microorganisms. The results of our pilot study on MHJL1 provide the theoretical basis and technical reference for improving the fermentation process and are conducive to the industrial development of the biocontrol capacity of the strain.

Based on the preliminary development of the liquid inoculant of strain MHJL1, research into the solid inoculant of strain MHJL1 was also tested (data not shown). Compared to traditional liquid microbial agents, solid microbial agents have advantages such as low cost, high content of viable spores, ease of storage, and convenience in transportation. The properties of the carriers play a key role in the quality of solid microbial agents [40,41]. For strain MHJL1, diatomaceous earth was chosen as the carrier, with polytetrafluoroethylene dispersion as the dispersant and dextrin as the UV protectant [42].

In addition to the function of preventing disease, *P. peoriae* also has growth-promoting functions in plants [43]. This study also first confirmed that strain MHJL1 had a good growth-promoting effect on cotton plants under different soil types. However, the exploration of the growth-promoting function of *P. peoriae* is still insufficient. The mechanisms of *P. peoriae* for plant growth and disease prevention still need to be discovered.

## 5. Conclusions

In this study, *P. peoriae* MHJL1 was isolated from the rhizosphere soil of cotton and was found to have antagonistic effects against fusarium and verticillium wilt while also improving the growth-promoting capacity of cotton seedlings. Sixteen gene clusters of secondary metabolites were identified from the genome of strain MHJL1, which can produce several fusaricidins to control pathogenic fungi. Improving the fermentation process enhanced the strain’s growth, spore production, and yield of antibacterial substances, which in turn boosted the disease-resistant growth ability of cotton seedlings.

## Figures and Tables

**Table 1:** Information about antimicrobial substances in the fermentation liquid of strain MHJL1.

Series of Fusaricidins	Theoretical Molecular Weight (Da)	Measured Molecular Weight (Da)	Retention Time/min	Peak Area (10^6^ mv·min)
LI-F04a (Fusaricidin A)	883.5611	883.5627	5.26	218.3836
LI-F04b (Fusaricidin B)/LI-F05a/LI-F06a	897.5768	897.5803	5.26	249.2986
LI-F03a (Fusaricidin C)	947.5560	947.5578	4.99	17.8481
LI-F03b (Fusaricidin D)	961.5717	961.5696	5.02	9.9679
LI-F05b/LI-F08a	911.5924	911.5931	5.44	140.2047
LI-F07a	931.5611	931.5644	5.51	312.9192
LI-F07b	945.5768	945.5735	5.72	2.8556

**Table 2:** Controlling the fusarium wilt and verticillium wilt of cotton by the microbial agent of strain MHJL1.

Soil Type	Treatment	Disease Indices (%)	Relative Efficacy (%)
Healthy Soil	*V. Dahliae*	61.64 ± 2.08 ^a^	-
	*V. Dahliae* + Strain MHJL1	25.72 ± 3.61 ^b^	58.27
	*F. Oxysporum*	55.28 ± 4.60 ^a^	-
	*F. Oxysporum* + Strain MHJL1	30.50 ± 1.28 ^b^	44.83
Continuously Cropped Soil	*V. Dahliae*	63.83 ± 2.72 ^a^	-
	*V. Dahliae* + Strain MHJL1	32.90 ± 1.68 ^b^	48.46
	*F. Oxysporum*	62.62 ± 2.69 ^a^	-
	*F. Oxysporum* + Strain MHJL1	28.04 ± 2.56 ^b^	55.22

Note: The data in the table are “mean ± standard deviations”, and groups with identical letters have no significant difference, while groups with different letters have a statistically significant difference.

**Table 3:** Comparison of cotton growth indicators under different set groups.

	Groups	Plant Height/cm	Root Length/cm	Stem Thickness/mm	Fresh Weight/g
Healthy Soil	Control	16.67 ± 1.54 ^d^	8.5 ± 2.59 ^c^	1.64 ± 0.28 ^c^	1.88 ± 0.33 ^c^
	Strain MHJL1	21.67 ± 1.03 ^a^	9.7 ± 0.57 ^a^	1.91 ± 0.07 ^a^	2.34 ± 0.17 ^a^
	*V. Dahliae*	15.93 ± 0.68 ^e^	5.83 ± 1.03 ^f^	1.49 ± 0.16 ^e^	1.35 ± 0.49 ^e^
	*V. Dahliae* + Strain MHJL1	19.93 ± 1.28 ^b^	8.77 ± 1.94 ^b^	1.70 ± 0.40 ^b^	2.01 ± 0.42 ^b^
	*F. Oxysporum*	12.63 ± 1.52 ^f^	6.1 ± 2.16 ^e^	1.34 ± 0.16 ^f^	1.07 ± 0.26 ^f^
	*F. Oxysporum* + Strain MHJL1	18.33 ± 1.84 ^c^	7.4 ± 1.61 ^d^	1.51 ± 0.26 ^d^	1.53 ± 0.28 ^d^
Continuously Cropped Soil	Control	13.23 ± 0.83 ^e^	8.17 ± 0.85 ^d^	1.65 ± 0.21 ^d^	0.89 ± 0.18 ^d^
	Strain MHJL1	17.4 ± 1.35 ^a^	10.97 ± 3.41 ^a^	2.12 ± 0.20 ^a^	1.96 ± 0.43 ^a^
	*V. Dahliae*	13.13 ± 0.72 ^f^	7.83 ± 1.21 ^e^	1.59 ± 0.13 ^e^	0.85 ± 0.19 ^e^
	*V. Dahliae* + Strain MHJL1	16.07 ± 0.75 ^b^	9.00 ± 1.18 ^b^	1.83 ± 0.21 ^b^	1.29 ± 0.16 ^b^
	*F. Oxysporum*	14.70 ± 0.82 ^d^	7.07 ± 0.68 ^f^	1.59 ± 0.05 ^e^	0.77 ± 0.10 ^f^
	*F. Oxysporum* + Strain MHJL1	14.83 ± 0.50 ^c^	8.80 ± 0.44 ^c^	1.72 ± 0.13 ^c^	1.12 ± 0.15 ^c^

Note: The data in the table are “mean ± standard deviations”, and groups with identical letters have no significant difference, while groups with different letters have a statistically significant difference.

## Data Availability

The complete genome sequence of strain MHJL1 was deposited in the NCBI GenBank database (accession number CP157265.1 and CP157266.1). Additional data can be provided on request. The original contributions presented in the study are included in the article; further inquiries can be directed to the corresponding author.

## Supplementary Materials

The following supporting information can be downloaded at: https://www.mdpi.com/article/10.3390/microorganisms13020261/s1, Figure S1: Effects of medium components on the number of living cells of strain MHJL1. Figure S2: The combination of response surface and regression equation analysis on the number of living cells of strain MHJL1 using different medium components. Figure S3: The effect of culture conditions on the number of living cells of strain MHJL1. Figure S4: The effect of medium components on the spore production of strain MHJL1. Figure S5: The combination of response surface and regression equation analysis on the spore production of strain MHJL1 using different medium components. Figure S6: The effect of culture conditions on the spore production of strain MHJL1. Figure S7: The combination of response surface and regression equation analysis on the spore production of strain MHJL1 under different culture conditions. Figure S8: The effect of medium components on the inhibition zone diameters of strain MHJL1 controlling *F. oxysporum* under different incubation time. Figure S9: The combination of response surface and regression equation analysis on the yield of antifungal substances of strain MHJL1 using different medium components.

## References

[1] Zhao Y., Jing H., Zhao P., Chen W., Li X., Sang X., Lü J., Wang H. (2021). GhTBL34 is associated with verticillium wilt resistance in cotton. Int. J. Mol. Sci..

[2] Liu H., Zeng Q., Yalimaimaiti N., Wang W., Zhang R., Yao J. (2021). Comprehensive genomic analysis of *Bacillus velezensis* AL7 reveals its biocontrol potential against *Verticillium* wilt of cotton. Mol. Genet. Genom..

[3] Zhang J., Abdelraheem A., Zhu Y., Heather D., Jane K.D., Derek P.W. (2022). Studies of evaluation methods for resistance to fusarium wilt race 4 (*Fusarium oxysporum* f. sp. *vasinfectum*) in cotton: Effects of cultivar, planting date, and inoculum density on disease progression. Front. Plant Sci..

[4] Liu L., Medison R.G., Zheng T., Meng X., Sun Z. (2023). Biocontrol potential of *Bacillus amyloliquefaciens* YZU-SG146 from *Fraxinus hupehensis* against *Verticillium* wilt of cotton. Biol. Control.

[5] Zhang Y., Zhou J., Zhao L., Feng Z. (2022). A review of the pathogenicity mechanism of *Verticillium dahliae* in cotton. J. Cotton Res..

[6] Gaspar Y.M., McKenna J.A., McGinness B.S., Hinch J., Poon S., Connelly A.A., Anderson M.A., Heath R.L. (2023). Field resistance to *Fusarium oxysporum* and *Verticillium dahliae* in transgenic cotton expressing the plant defensin NaD1. J. Exp. Bot..

[7] Klosterman S.J., Atallah Z.K., Vallad G.E., Subbarao K.V. (2009). Diversity, pathogenicity, and management of verticillium species. Annu. Rev. Phytopathol..

[8] Garbeva P., Hol W.G., Termorshuizen A.J., Kowalchuk G.A., De Boer W. (2011). Fungistasis and general soil biostasis—A new synthesis. Soil Biol. Biochem..

[9] Li R., Tao R., Ling N., Chu G. (2017). Chemical, organic and bio-fertilizer management practices effect on soil physicochemical property and antagonistic bacteria abundance of a cotton field: Implications for soil biological quality. Soil Till. Res..

[10] Jung T., Kim J.H., Song H.G. (2012). Antifungal activity and plant growth promotion by rhizobacteria inhibiting growth of plant pathogenic fungi. Korean J. Microbiol..

[11] Ricardo A., Christopher D., Christopher M.M. (2020). Analogous wheat root rhizosphere microbial successions in field and greenhouse trials in the presence of biocontrol agents *Paenibacillus peoriae* SP9 and *Streptomyces fulvissimus* FU14. Mol. Plant Pathol..

[12] Olishevska S., Nickzad A., Restieri C., Dagher F., Luo Y., Zheng J., Eric D. (2023). *Bacillus velezensis* and *Paenibacillus peoriae* strains effective as biocontrol agents against *Xanthomonas* bacterial spot. Appl. Microbiol..

[13] Yuan L., Jiang H., Jiang X., Li Y., Lu P., Yin X., Wei Y. (2022). Comparative genomic and functional analyses of *Paenibacillus peoriae* ZBSF16 with biocontrol potential against grapevine diseases, provide insights into its genes related to plant growth-promoting and biocontrol mechanisms. Front. Microbiol..

[14] Jiang A., Zou C., Xu X., Ke Z., Hou J., Jiang G., Fan C., Gong J., Wei J. (2022). Complete genome sequence of biocontrol strain *Paenibacillus peoriae* HJ-2 and further analysis of its biocontrol mechanism. BMC Genom..

[15] Li W., Tao Y., Zhao S., Niu Q. (2020). Antagonism of *Bacillus* sp. HT-7 against verticillium wilt of cotton and exploration of antagonistic factors. J. Henan Agric. Sci..

[16] Zhang D., Ren L., Wang Q., Li W., Song Z., Jin X., Fang W., Yan D., Li Y., Wang Q. (2024). Systematic assessment of the antifungal mechanism of soil fumigant methyl isothiocyanate against *Fusarium oxysporum*. Environ. Pollut..

[17] Sun Y., Wang C., Wang X., Du B., Liu K., Wang C. (2024). Biocontrol characteristics of *Bacillus atrophaeus* CNY01 and its salt-resistant and growth-promoting effect on maize seedling. Biotechnol. Bull..

[18] Xu Y., Yu Y., Tian Y., Su Y., Li X., Zhang Z., Zhu H., Han J., Zhang H., Liu L. (2018). Analysis of Beijing Douzhir Microbiota by High-Throughput Sequencing and Isolation of Acidogenic, Starch-Flocculating Strains. Front. Microbiol..

[19] Wan C., Liu B., Chen M. (2018). Chemical constituents and antifungal activity of *Paenibacillus brasilensis* YS-1. Mol. Plant Breed.

[20] Wang C., Pei J., Li H., Zhu X., Zhang Y., Wang Y., Li W., Wang Z., Liu K., Du B. (2024). Mechanisms on salt tolerant of *Paenibacillus polymyxa* SC2 and its growth-promoting effects on maize seedlings under saline conditions. Microbiol. Res..

[21] Yao Z., Chen Y., Luo S., Wang J., Zhang J., Zhang J., Tian C., Tian L. (2022). Culturable screening of plant growth-promoting and biocontrol bacteria in the rhizosphere and phyllosphere of wild rice. Microorganisms.

[22] Xiao G., He P., Zhao P., Liu H., Zhang L., Pang C., Yu J. (2018). Genome-wide identification of the *GhARF* gene family reveals that *GhARF2* and *GhARF18* are involved in cotton fibre cell initiation. J. Exp. Bot..

[23] Zhang P., Jiang D., Wang Y., Yao X., Luo Y., Yang Z. (2021). Comparison of be novo assembly strategies for bacterial genomes. Int. J. Mol. Sci..

[24] Koren S., Walenz B.P., Berlin K., Miller J.R., Bergman N.H., Phillippy A.M. (2017). Canu: Scalable and accurate long-read assembly via adaptive k-mer weighting and repeat separation. Genome Res..

[25] Walker B.J., Abeel T., Shea T., Priest M., Abouelliel A. (2017). Pilon: An integrated tool for comprehensive microbial variant detection and genome assembly improvement. PLoS ONE.

[26] Galperin M.Y., Makarova K., Wolf Y., Koonin E.V. (2015). Expanded microbial genome coverage and improved protein family annotation in the COG database. Nucleic Acids Res..

[27] Krzywinski M., Schein J., Birol I., Connors J., Gascoyne R., Horsman D., Jones S.J., Marra M.A. (2009). Circos: An information aesthetic for comparative genomics. Genome Res..

[28] Deng Y., Lu Z., Bi H., Lu F., Zhang C., Bie X. (2011). Isolation and characterization of peptide antibiotics LI-F04 and polymyxin B 6 produced by *Paenibacillus polymyxa* strain *JSa-9*. Peptides.

[29] Li J., Zhang L., Ye G., Zhu L., Lin J., Wang C., Du B., Ding Y., Mei X. (2022). Synergistic effect of co-culture rhizosphere *Streptomyces*: A promising strategy to enhance antimicrobial activity and plant growth-promoting function. Front. Microbiol..

[30] Stancliffe E., Schwaiger-Haber M., Sindelar M., Patti G.J. (2021). DecoID improves identification rates in metabolomics through database-assisted MS/MS deconvolution. Nat. Methods.

[31] Breig S.J.M., Luti K.J.K. (2021). Response surface methodology: A review on its applications and challenges in microbial cultures. Mater. Today Proc..

[32] Wang Y., Zhang F., Wang C., Guo P., Han Y., Zhang Y., Sun B., Shan S., Ruan W., Pan J. (2022). Antifungal substances produced by *Xenorhabdus bovienii* and its inhibition mechanism against *Fusarium solani*. Int. J. Mol. Sci..

[33] Li N., Chen S., Yan Z., Han J., Ta Y., Pu T., Wang Y. (2021). Antimicrobial activity and identification of the biosynthetic gene cluster of X-14952B from *Streptomyces* sp. 135. Front. Microbiol..

[34] Zhao Y., Xie X., Li J., Shi Y., Chai A., Fan T., Li B., Li L. (2022). Comparative genomics insights into a novel biocontrol agent *Paenibacillus peoriae* strain ZF390 against bacterial soft rot. Biology.

[35] Raj Y.D., Mahesh A., Sang W.K., Kin H.S., Lee Y.S. (2021). Suppression of fusarium wilt caused by *Fusarium oxysporum* f. sp. *lactucae* and growth promotion on lettuce using bacterial isolates. J. Microbiol. Biotechnol..

[36] Langendries S., Goormachtig S. (2021). *Paenibacillus polymyxa*, a Jack of all trades. Environ. Microbiol..

[37] Stephen A.C., John C.V. (2016). Lipopeptides from *Bacillus* and *Paenibacillus* spp.: A Gold Mine of Antibiotic Candidates. Med. Res. Rev..

[38] Cochrane S.A., Vederas J.C. (2014). Unacylated tridecaptin A_1_ acts as an effective sensitiser of Gram-negative bacteria to other antibiotics. Int. J. Antimicrob. Agents.

[39] Sa R., Sun Y., Cao Y., Yan W., Zong Z., An W., Song M. (2024). Medium optimization and fermentation kinetics for antifungal compounds production by an endophytic *Paenibacillus polymyxa* DS-R5 isolated from *Salvia miltiorrhiza*. Curr. Microbiol..

[40] Arora A., Nain L., Gupta J.K. (2005). Solid-state fermentation of wood residues by *Streptomyces griseus* B1, a soil isolate, and solubilization of lignins. World J. Microbiol. Biotechnol..

[41] El-Hassan S.A., Gowen S.R. (2006). Formulation and delivery of the bacterial antagonist *Bacillus subtilis* for management of lentil vascular wilt caused by *Fusarium oxysporum* f. sp. *lentis*. J. Phytopathol..

[42] Hua C., Li L., Juan H., Yuan H., Cheng S. (2015). A preliminary preparation of endophytic bacteria CE3 wettable powder for biological control of postharvest diseases. Not. Bot. Horti Agrobot..

[43] Wang K., Lin Z., Dou J., Jiang M., Shen N., Feng J. (2023). Identification and surveys of promoting plant growth VOCs from biocontrol bacteria *Paenibacillus peoriae* GXUN15128. Microbiol. Spectr..

