# Supplementary material for: Identification, Genome Characterization, and Growth Optimization of Paenibacillus peoriae MHJL1 for Biocontrol and Growth Promotion of Cotton Seedlings
Source: Microorganisms. 2025 Jan 24;13(2):261. doi: 10.3390/microorganisms13020261 (PMC11857793; doi:10.3390/microorganisms13020261)
Supplement: Supplementary file 1 [file microorganisms-13-00261-s001.zip › microorganisms-3400177-supplementary.pdf]

Supplementary material

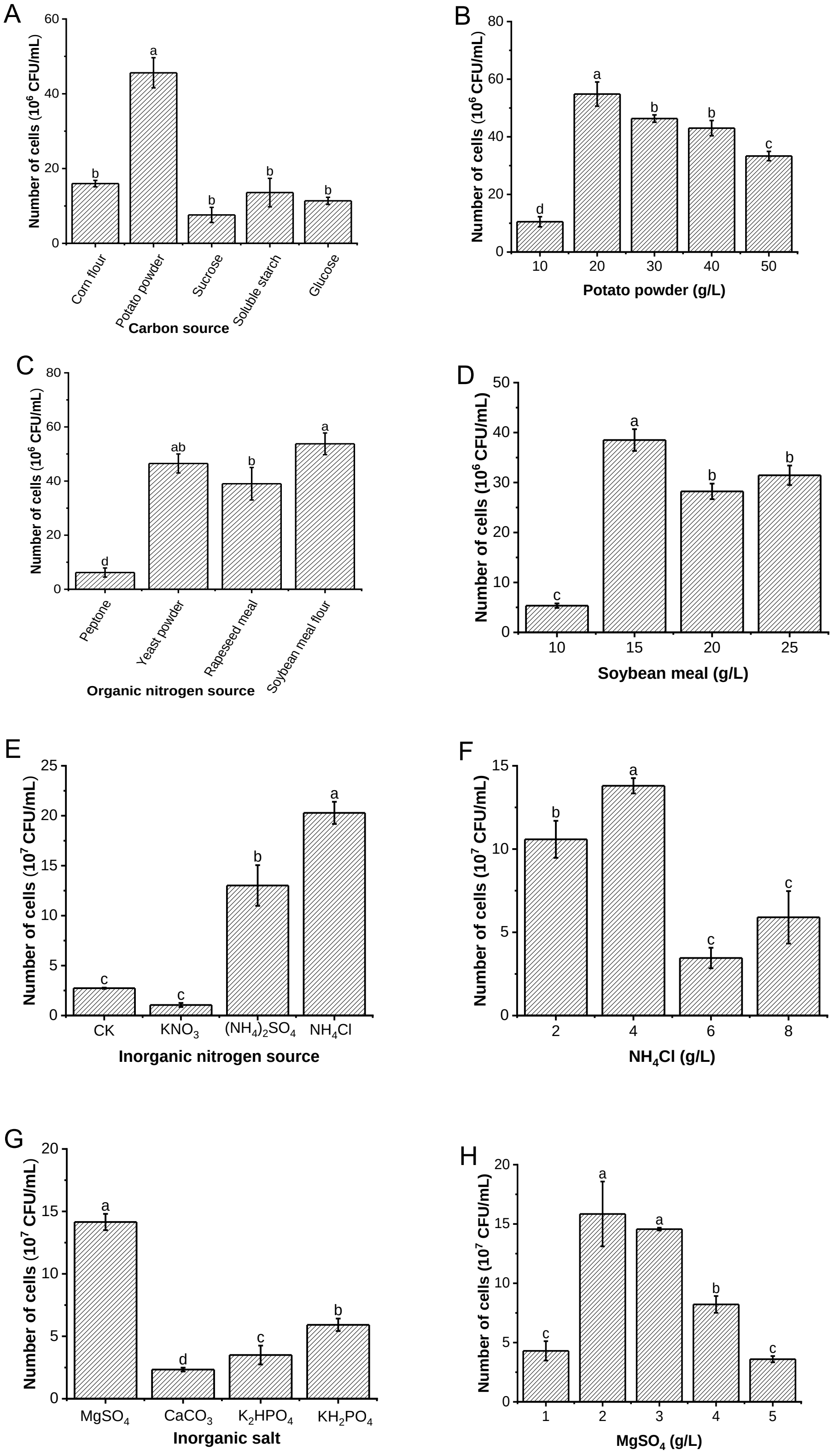

Figure S1. Effects of medium components on the number of living cells of strain MHJL1. (A) Effects of different carbon sources on the number of living cells of strain MHJL1. (B) Effects of different concentrations of potato powder on the number of

living cells of strain MHJL1. (C) Effects of different organic nitrogen sources on the number of living cells of strain MHJL1. (D) Effects of different concentrations of soybean meal on the number of living cells of strain MHJL1. (E) Effects of different inorganic nitrogen sources on the number of living cells of strain MHJL1. (F) Effects of different concentrations of NH_4Cl on the number of living cells of strain MHJL1. (G) Effects of different inorganic salts on the number of living cells of strain MHJL1. (H) Effects of different concentrations of $MgSO_4$ on the number of living cells of strain MHJL1. Letter-based method was used for indicating significant differences. Groups with identical letter denote no significant difference, while groups with different letter indicate a statistically significant difference.

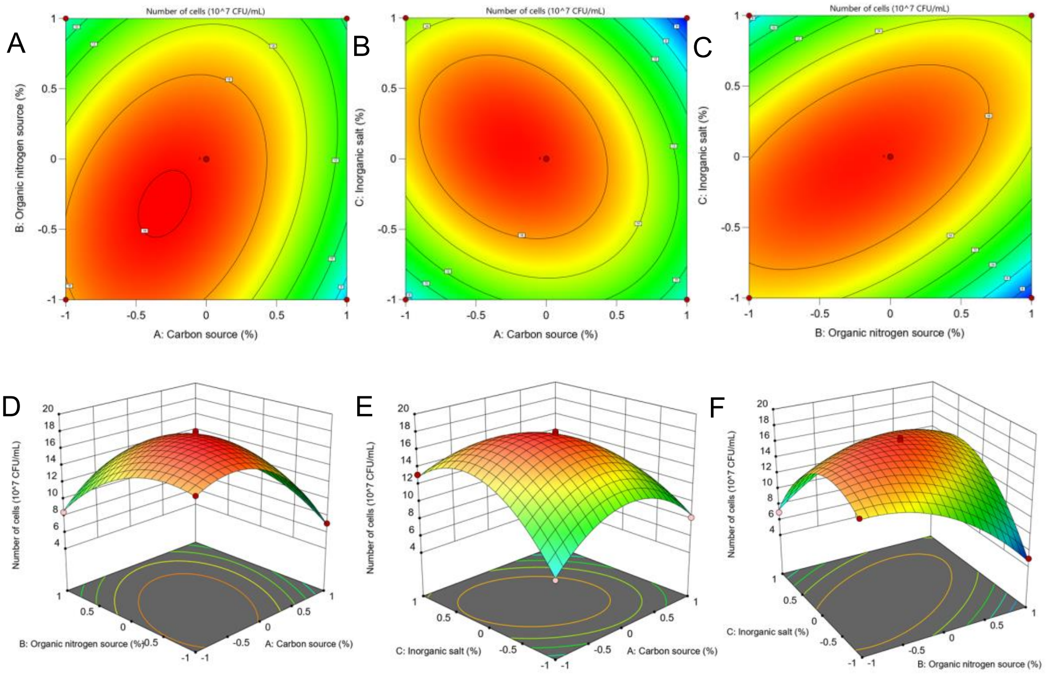

Figure S2. The combination of response surface and regression equation analysis on the number of living cells of strain MHJL1 using different medium components. (A) and (D) represent the response surface and contour plot of the interaction between carbon source A (potato powder) and organic nitrogen source B (soybean meal) on the number of living cells of strain MHJL1, respectively. (B) and (E) represent the response surface and contour plot of the interaction between carbon source A (potato powder) and inorganic salt C ($MgSO_4$) on the number of living cells of strain MHJL1, respectively. (C) and (F) represent the response surface and contour plot of the interaction between organic nitrogen source B (soybean meal) and inorganic salt C ($MgSO_4$) on the number of living cells of strain MHJL1, respectively.

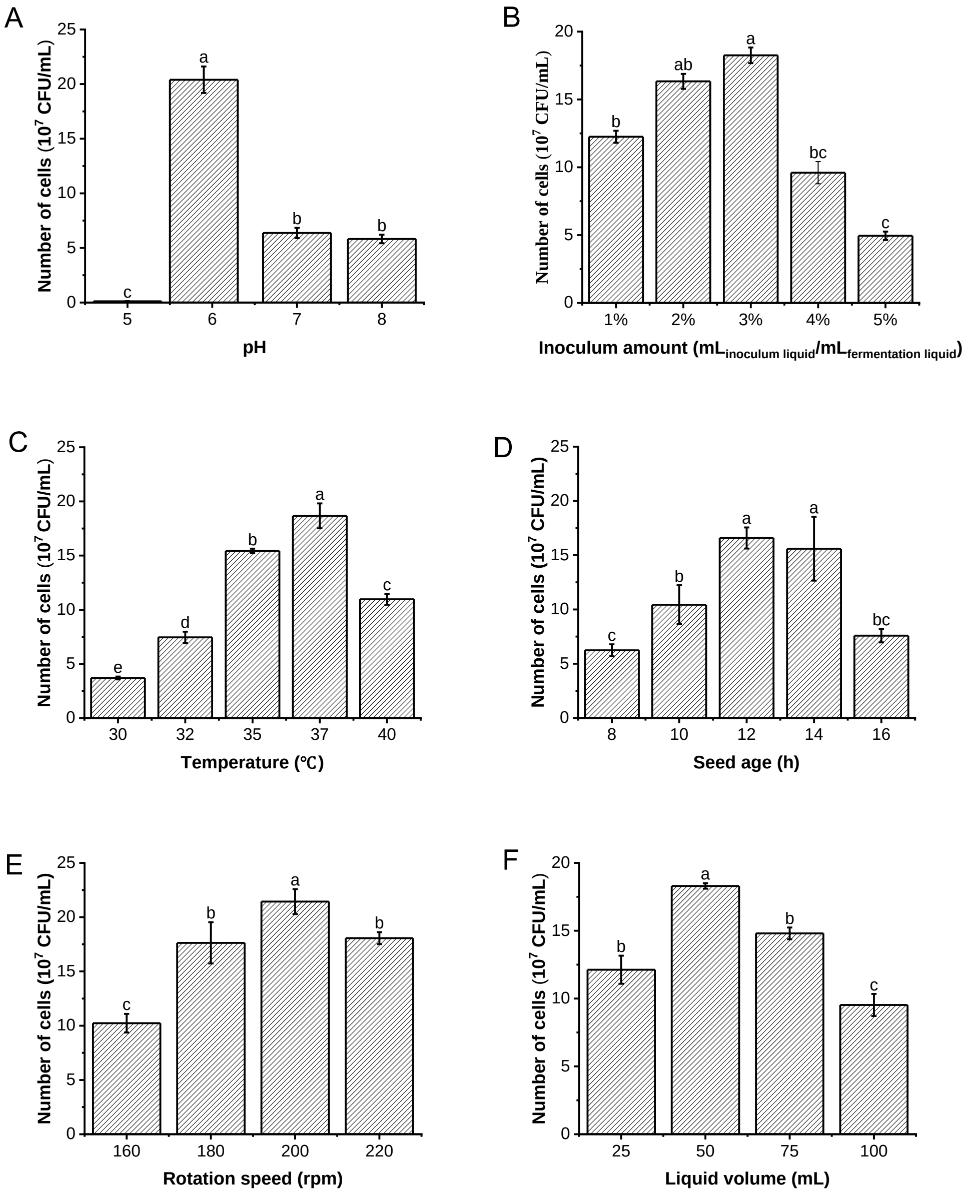

Figure S3. The effect of culture conditions on the number of living cells of strain MHJL1. (A) The effect of different pH levels on the number of living cells of strain MHJL1. (B) The effect of different inoculation amounts on the number of living cells of strain MHJL1. (C) The effect of different temperatures on the number of living cells of strain MHJL1. (D) The effect of different ages of strains on the number of living cells of strain MHJL1. (E) The effect of different rotation speeds on the number of living cells of strain MHJL1. (F) The effect of different liquid volumes on the number of living cells of strain MHJL1. The volume of flasks used in the experiment is 250 mL. Letter-based method was used for indicating significant differences. Groups with identical letter denote no significant difference, while groups with

different letter indicate a statistically significant difference.

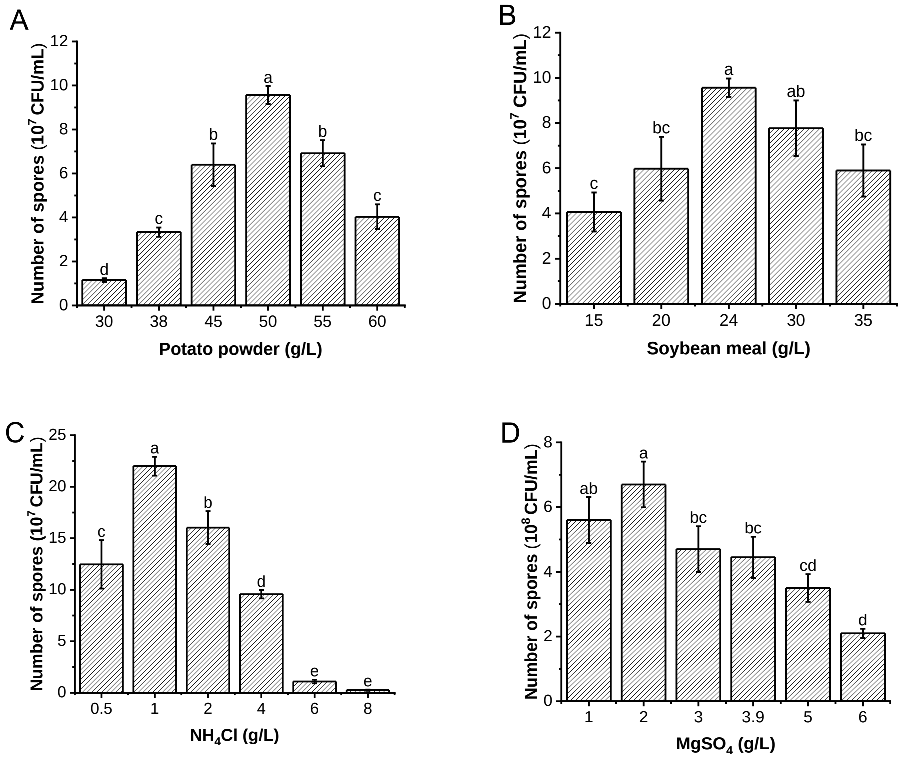

Figure S4. The effect of medium components on the spore production of strain MHJL1. (A) The effect of different concentrations of potato powder on the spore production of strain MHJL1. (B) The effect of different concentrations of soybean meal powder on the spore production of strain MHJL1. (C) The effect of different concentrations of NH_4Cl on the spore production of strain MHJL1. (D) The effect of different concentrations of $MgSO_4$ on the spore production of strain MHJL1. Letter-based method was used for indicating significant differences. Groups with identical letter denote no significant difference, while groups with different letter indicate a statistically significant difference.

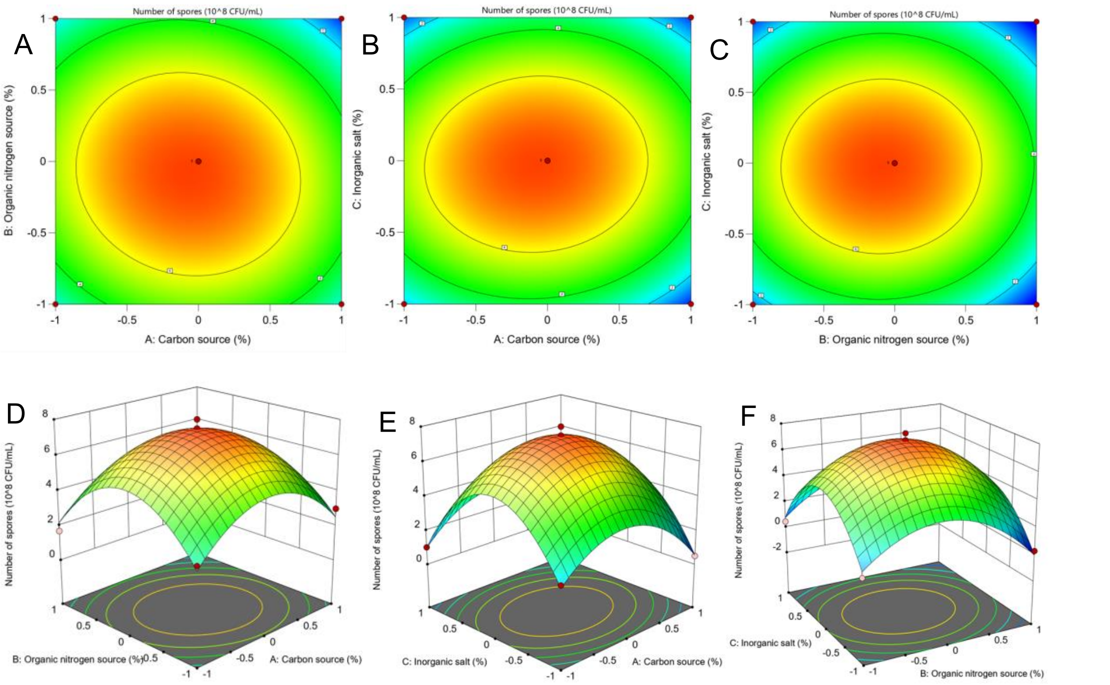

Figure S5. The combination of response surface and regression equation analysis on the spore production of strain MHJL1 using different medium components. (A) and (D) represent the response surface and contour plot of the interaction between carbon source A (potato starch) and organic nitrogen source B (soybean meal) on the sporulation of strain MHJL1 in fermentation broth, respectively. (B) and (E) represent the response surface and contour plot of the interaction between carbon source A (potato starch) and inorganic salt C ($MgSO_4$) on the sporulation of strain MHJL1 in fermentation broth, respectively. (C) and (F) represent the response surface and contour plot of the interaction between organic nitrogen source B (soybean meal) and inorganic salt C ($MgSO_4$) on the sporulation of strain MHJL1 in fermentation broth, respectively.

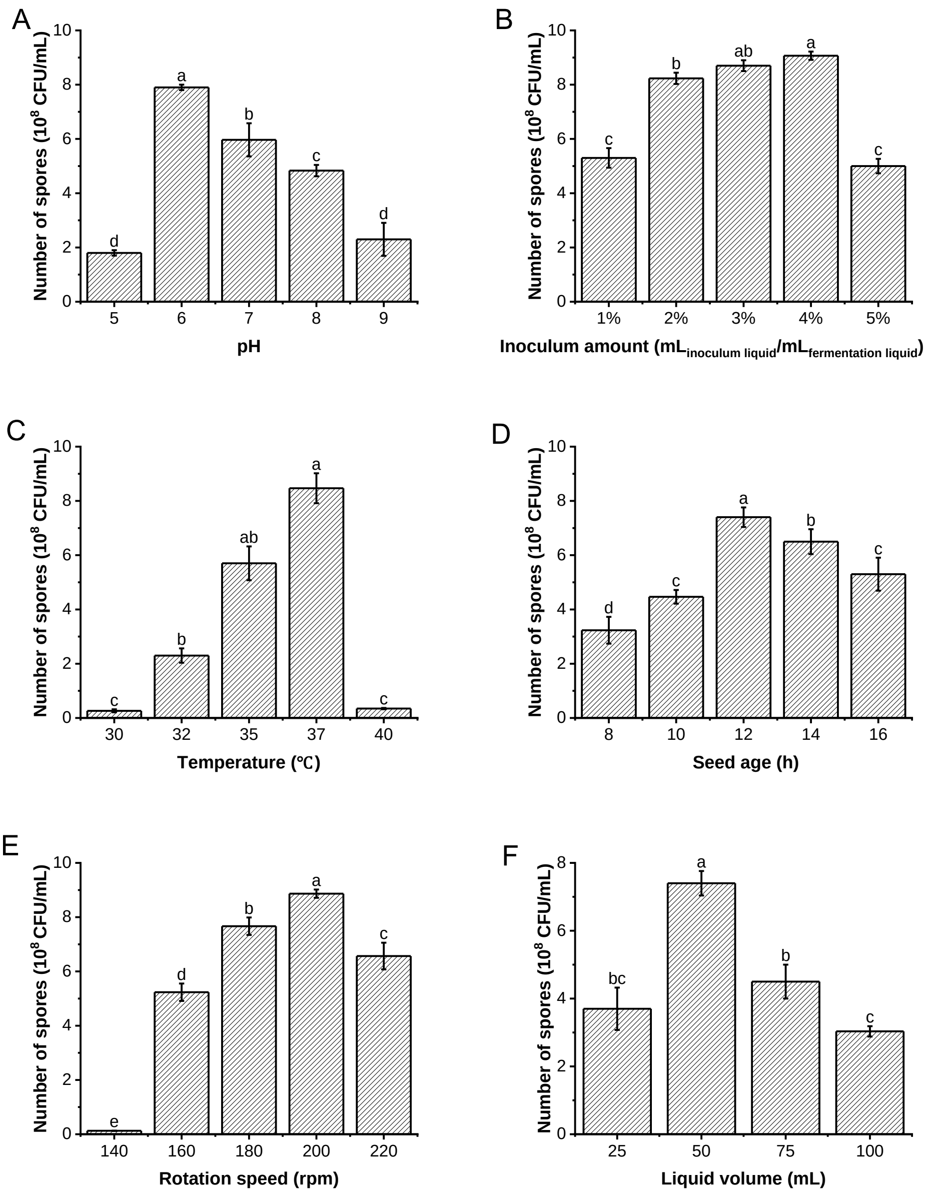

Figure S6. The effect of culture conditions on the spore production of strain MHJL1. (A) The effect of different pH levels on the spore production of strain MHJL1. (B) The effect of different inoculation amounts on the spore production of strain MHJL1. (C) The effect of different temperatures on the spore production of strain MHJL1. (D) The effect of different ages of strains on the spore production of strain MHJL1. (E) The effect of different rotation speeds on the spore production of strain MHJL1. (F) The effect of different liquid volumes on the spore production of strain MHJL1. The volume of the flasks used in the experiment is 250 mL. Letter-based method was used for indicating significant differences. Groups with identical letter denote no

significant difference, while groups with different letter indicate a statistically significant difference.

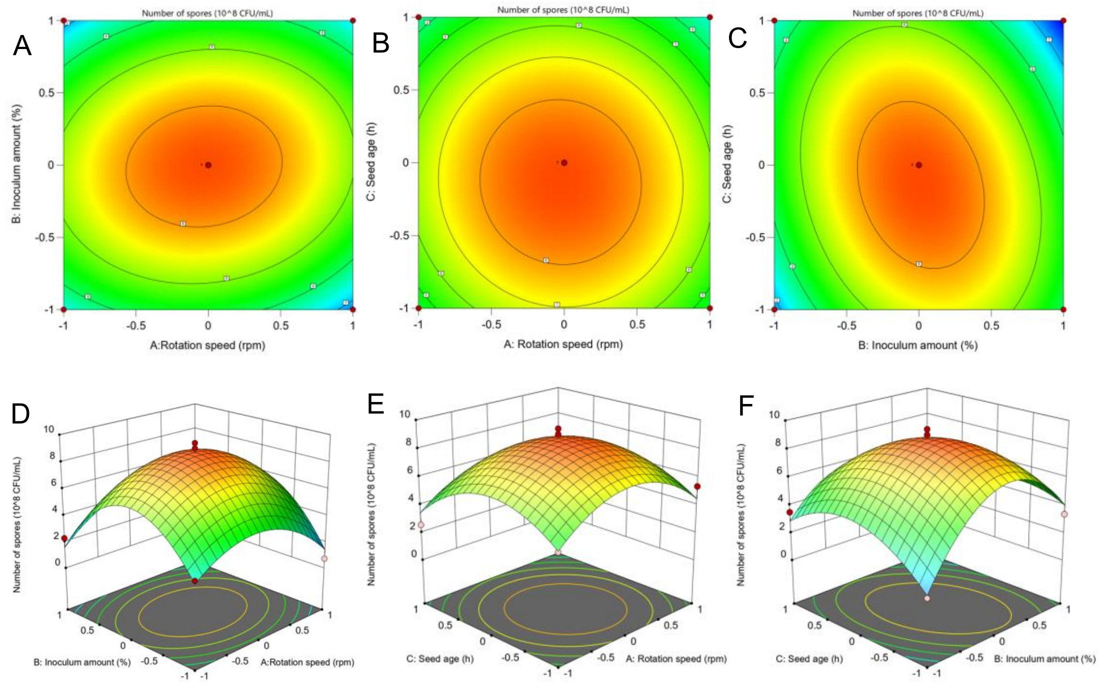

Figure S7. The combination of response surface and regression equation analysis on the spore production of strain MHJL1 under different culture conditions. (A) and (D) represent the response surface and contour plot of the interaction between rotation speed A and inoculum amount B on the spore production of strain MHJL1, respectively. (B) and (E) represent the response surface and contour plot of the interaction between rotation speed A and seed age C on the spore production of strain MHJL1, respectively. (C) and (F) represent the response surface and contour plot of the interaction between inoculum amount B and seed age C on the spore production of strain MHJL1, respectively.

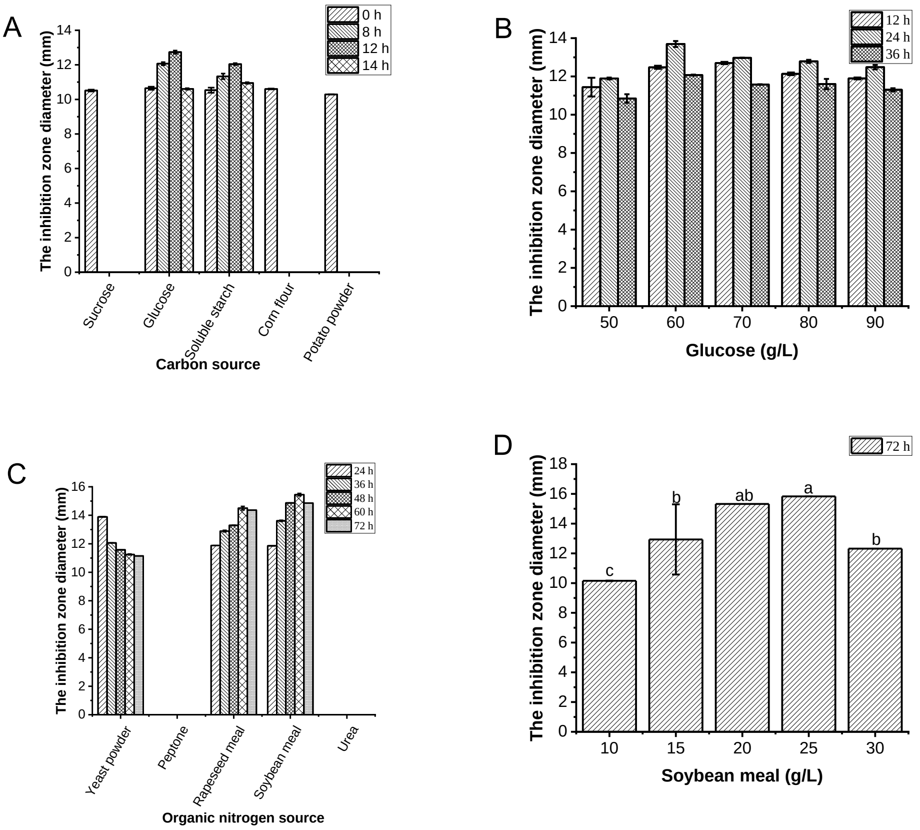

Figure S8. The effect of medium components on the inhibition zone diameters of strain MHJL1 controlling *F. oxysporum* under different incubation time. (A) The effect of different carbon source components on the yield of antifungal substances of strain MHJL1. (B) The effect of different concentrations of glucose on the yield of antifungal substances of strain MHJL1. (C) The effect of different organic nitrogen source components on the yield of antifungal substances of strain MHJL1. (D) The effect of different concentrations of soybean meal powder on the yield of antifungal substances of strain MHJL1. Letter-based method was used for indicating significant differences. Groups with identical letter denote no significant difference, while groups with different letter indicate a statistically significant difference.

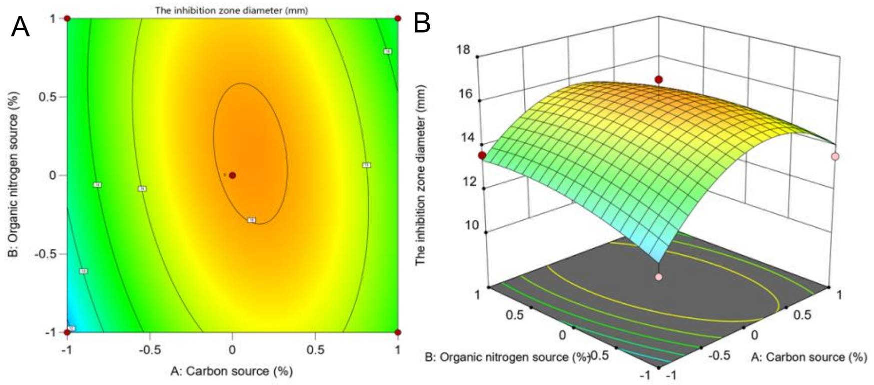

Figure S9. The combination of response surface and regression equation analysis on the yield of antifungal substances of strain MHJL1 using different medium components. (A) and (B) represent the response surface and contour plot of the interaction between carbon source A (glucose) and organic nitrogen source B (soybean meal) on the yield of antifungal substances of strain MHJL1, respectively.
